# Distribution of *Barley yellow dwarf virus*-PAV in the Sub-Antarctic Kerguelen Islands and Characterization of Two New *Luteovirus* Species

**DOI:** 10.1371/journal.pone.0067231

**Published:** 2013-06-18

**Authors:** Laurence Svanella-Dumas, Thierry Candresse, Maurice Hullé, Armelle Marais

**Affiliations:** 1 INRA, UMR 1332 de Biologie du Fruit et Pathologie, CS20032 Villenave d′Ornon, France; 2 Univ. Bordeaux, UMR 1332 de Biologie du Fruit et Pathologie, CS20032 Villenave d′Ornon, France; 3 Institut de Génétique, Environnement et Protection des Plantes, Agrocampus Rennes, UMR INRA 1349, BP 35327, Le Rheu, France; University of California, Riverside, United States of America

## Abstract

A systematic search for viral infection was performed in the isolated Kerguelen Islands, using a range of polyvalent genus-specific PCR assays. *Barley yellow dwarf virus* (BYDV) was detected in both introduced and native grasses such as *Poa cookii*. The geographical distribution of BYDV and its prevalence in *P. cookii* were analyzed using samples collected from various sites of the archipelago. We estimate the average prevalence of BYDV to be 24.9% in *P. cookii*, with significant variability between sites. BYDV genetic diversity was assessed using sequence information from two genomic regions: the P3 open reading frame (ORF) (encoding the coat protein) and the hypervariable P6 ORF region. The phylogenetic analysis in the P3 region showed that BYDV sequences segregate into three major lineages, the most frequent of which (Ker-I cluster) showed close homology with BYDV-PAV-I isolates and had very low intra-lineage diversity (0.6%). A similarly low diversity was also recorded in the hypervariable P6 region, suggesting that Ker-I isolates derive from the recent introduction of BYDV-PAV-I. Divergence time estimation suggests that BYDV-PAV-I was likely introduced in the Kerguelen environment at the same time frame as its aphid vector, *Rhopalosiphum padi*, whose distribution shows good overlap with that of BYDV-Ker-I. The two other lineages show more than 22% amino acid divergence in the P3 region with other known species in the BYDV species complex, indicating that they represent distinct BYDV species. Using species-specific amplification primers, the distribution of these novel species was analyzed. The high prevalence of BYDV on native *Poaceae* and the presence of the vector *R. padi*, raises the question of its impact on the vulnerable plant communities of this remote ecosystem.

## Introduction

Because of their isolation, restricted biota and harsh environments, Antarctic and sub-Antarctic terrestrial ecosystems are relatively simple, but highly vulnerable to biotic and abiotic perturbations. The French sub-Antarctic islands represent an original and constrained ecosystem characterized by a paucispecific flora consisting of mostly endemic species. In particular, the Kerguelen Islands (7215 km^2^), which comprise the second most isolated archipelago on earth, are located close to the Antarctic Polar Frontal Zone (APFZ), a major oceanic and climatic boundary. They constitute an exceptional site as biological monitoring has been performed there for more than 35 years, providing an excellent knowledge of their paucispecific fauna and flora which are mostly distributed in a limited altitudinal range along the coasts [1]. These studies concerned the abundance, phenology, and distribution of native and introduced species [2] as well as community dynamics in a changing environment [3], [4]. This isolated ecosystem is particularly at risk, both through the effect of global climate change and through further introduction of invading species, including those of plant viruses and/or of their vectors [5]. Particularly relevant for plant viruses is the fact that only five aphid species are known to be present in the Kerguelen archipelago (only three species in the wild, outside of glasshouses) [6] and that all of them appear to be relatively recent introductions during the second half of the 20th century (Voisin, personal communication, and [7]).

Unfortunately, our knowledge and understanding of the prevalence, distribution and impact of plant viruses in such remote and constrained ecosystems and, more generally, in natural plant communities, can be considered skeletal at best. This situation largely results from the fact that the efforts of the plant virology community have very largely been concentrated on crop plants and on the viruses that affect them [8], [9], [10], [11]. In Antarctic and sub-Antarctic ecosystems, very little is known on plant virus biodiversity or even presence, with the exception of the description of a new *Badnavirus* from Macquarie Island [12], and the report of the presence of well-known viruses such as *Cherry leaf roll virus* (CLRV), detected on introduced Nasturtium plants on Amsterdam Island [13]. Preliminary results of a study in the Argentina Islands reported the detection in the Antarctic hairgrass *Deschampsia antarctica* of three well known and widespread plant viruses, *Cucumber mosaic virus* (CMV, *Cucumovirus*), *Cucumber green mottle mosaic virus* (CGMMV, *Tobamovirus*), and *Tomato spotted wilt virus* (TSWV, *Tospovirus*) [14]. However these results were only based on serological tests so that the unambiguous identification of these agents still remains to be established.

With the aim of gaining a better perspective on plant virus ecology in a sub-Antarctic ecosystem, an initial screening of a limited number of plant samples collected in the Kerguelen Islands was performed using a range of polyvalent, virus genus-specific PCR assays [15]. Preliminary results indicated the presence of *Barley yellow dwarf virus* (BYDV) on a native grass, *Poa cookii* (Cook's tussock-grass or bluegrass). BYDV is a grass- and cereal-infecting virus of worldwide distribution and of major economic significance [16]. It is the type member of the genus *Luteovirus*, in the family *Luteoviridae* [17] and, as with other Luteoviruses, it is transmitted only by aphids in a persistent fashion and is not mechanically transmissible. BYDV is also not known to be seed transmitted [16]. Initially described as a single virus species encompassing several strains differentiated by serological properties and by their aphid vector species specificities, BYDV is now recognized as a complex of species. On the basis of their different genome structure, some BYDV strains have been renamed as *Cereal yellow dwarf virus* (two species, CYDV-RPV and CYDV-RPS) in the genus *Polerovirus* while others (BYDV-SGV, -GPV, -RMV) are currently unassigned to any genus in the family [17], [18]. Those strains remaining in the genus *Luteovirus* have acquired an independent species status based on differences in vector specificity and in serology (BYDV-PAV, BYDV-MAV, BYDV-PAS, tentatively BYDV-GAV). The currently accepted sequence-based species demarcation criterion is more than 10% amino acid sequence divergence in any of the viral gene products [17]. The situation is however further complicated by the pervasive role of recombination events in the evolution of BYDV [18], [19] and by the fact that high sequence divergence between isolates has led to the description of several species or subspecies within BYDV-PAV, named respectively BYDV-PAV-I, BYDV-PAV-II (formerly BYDV-PAS) and PAV-IIIa/IIIb [18], [20].

The presence of BYDV in the Kerguelen Islands is interesting on several counts. First, it is surprising to encounter such a strictly aphid-vectored virus in an environment that was devoid of any aphid species until recent times (Voisin, unpublished data, and [7]), strongly suggesting that BYDV is itself a recent introduction and warranting an investigation of the isolates BYDV present. Second, BYDV has been reported in some environments to contribute to competition between native and introduced grass species by increasing the pathogen load of susceptible native species with which the introduced, more tolerant, grasses compete. [21], [22], [23]. BYDV could therefore have the potential to cause similar detrimental effects in the context of Kerguelen Islands environment, where the number of introduced plants is higher than the number of native ones.

In the present work we analysed the distribution, prevalence and diversity of BYDV on Kerguelen Islands. The results show that BYDV-PAV-I is the most prevalent species and that it is much more prevalent in the native *P. cookii*, although it has been detected in a few other native and introduced grasses, but at a much lower rate. Two other highly divergent BYDV-like viruses were also identified, likely representing novel species.

## Materials and Methods

### Virus isolates sampling and aphid surveys

All vegetation sampling in the Kerguelen Islands was performed under permits 2008-97, 2009-51, and 2010-76 issued by the relevant TAAF (Terres Australes et Antarctiques Françaises) Authorities.

BYDV isolates were collected during the summer campaigns 2008–2009, 2009–2010, and 2010–2011 from *P. cookii* (Table 1) and from other native or introduced grasses listed in Table 2.

**Table 1:** Detection of *Barley yellow dwarf virus* in *Poa cookii* sampled at various Kerguelen Islands sites and concomitant presence of *Rhopalosiphum padi*.

Prospected sites	Collection site	BYDV infected/tested^a^	Presence of *R. padi* b
Presqu'île Ronarc'h	P12	2/19	0
	Phonolite	0/5	0
Ile Longue	Long6	5/15	1
	Long20	1/7	1
Observatoire		1/1	nt
Ile Guillou		1/1	0
Ile Australia	Aus7	14/19	2
	Aus16	2/11	nt
Ile Mayes		11/11	1
Péninsule Courbet	TC5	0/18	0
	TC7	0/9	0
	TC8	0/5	0
	TC9	0/5	0
	TC10	0/5	0
	TC11	0/20	0
	TC13	5/5	0
	TC15	0/18	0
	TC17	4/5	0
	TC18	0/10	0
	TC19	3/12	2
Presqu'ile du Prince de Galles	Pointe Suzanne	4/6	2
	Chionis	37/101	1–2^c^
Anse du Cartographe		0/11	0
Presqu'île Jeanne d′Arc	Sourcils Noirs	0/9	nt
Val Studer		0/1	0
Ile du Port		0/2	nt
Port Perrier		0/12	0
Anse Ring	AR1	0/2	0
	AR2	0/1	0
	AR3	0/1	0
Ile aux Moules		1/1	nt
Ile du Canard		0/1	nt
Ile aux Skuas		1/1	nt
Ile Bryer		2/5	nt
Baie de la Mouche		0/1	0
Vallée des Sables		0/1	0
Cap Français		0/1	0
Baie de l′Oiseau		0/5	0
BaieDucheyron		0/5	0
Port Matha		0/5	0
Ile Bethell		0/5	0

a Results are a combination of immune tissue printing and RT-PCR for detection of BYDV.

b the abundance of *R. padi* is indicated using the following code: 0 = not observed; 1 = present in low numbers; 2 = locally abundant; nt: missing data.

c abundance of *R. padi* variable depending of the observation year.

**Table 2:** Detection of *Barley yellow dwarf virus* in various grass species in the Kerguelen Islands and prevalence of *Rhopalosiphum padi* on these species.

Species^a^	Native/Introduced	*R. padi* b	BYDV infected/tested^c^
*Agrostis capillaris* (p)	Introduced	1	0/6
*Agrostis magellanica* (p)	Native	nt	1/25
*Alopecurus geniculatus* (p)	Introduced	2	0/5
*Alopecurus pratensis* (p)	Introduced	nt	0/2
*Anthoxanthum odoratum* (p)	Introduced	0	0/1
*Arrhenatherum elatius* (p)	Introduced	0	0/3
*Dactylis glomerata* (p)	Introduced	1	1/19
*Deschampsia antartica* (p)	Native	1	0/15
*Festuca contracta* (p)	Native	2	2/26
*Festuca pratensis* (p)	Introduced	nt	0/2
*Festuca rubra* (p)	Introduced	1	0/4
*Holcus lanatus* (p)	Introduced	2	0/3
*Lolium perene* (p)	Introduced	nt	0/3
*Phleum pratense* (p)	Introduced	nt	0/3
*Poa annua* (b)	Introduced	2	2/33
*Poa cookii* (p)	Native	2	94/378
*Poa kerguelensis* (p)	Native	0	0/14
*Poa nemoralis* (p)	Introduced	nt	0/2
*Poa pratensis* (p)	Introduced	1	0/14
*Poa trivialis* (p)	Introduced	1	0/3
*Vulpia bromoides* (a)	Introduced	0	0/9

a The status of the various grasses is indicated in parentheses: p: perennial, a: annual, b: biennial. Data compiled from Lebouvier (personal communication) and Frenot et al. [1]

b the prevalence of *R. padi* on the various grasses is indicated using the following code: 0 = not observed; 1 = present in low numbers; 2 = locally abundant, and nt missing data. Data compiled from Hullé et al. [6]

c BYDV was detected by RT-PCR unless for *Poa cookii* for which the results are a combination of immune tissue printing and RT-PCR.

Generally, random sampling was performed but grasses species showing yellowing or reddening symptoms reminiscent of BYDV infection were systematically collected in sites rich in *P. cookii*. All collection points were identified by GPS (listed on Table S1). For data analysis, collection sites within a two kilometer radius were considered as representing a single sampling point. Leaves and stem for each collected plant were dried in a paper bag containing granular anhydrous calcium chloride (Sigma, cat. no. C1016).

The spatial distribution and host plant range of aphids were established during several summer campaigns from 2001 to 2011. The abundance was estimated as often and at as many locations as possible. On each occasion, the abundance of aphids (3 classes of abundance) was estimated by searching for aphids on the same plant species during 30 min in 2001 [6] and 15 min during the following years. Aphids were searched by beating plants of all size above a white tray with an average 30–40 beating per 15 min within a 200 to 1000 m^2^ area depending on plant distribution and abundance.

### Detection of BYDV-PAV and BYDV-MAV by immunoprinting

A first evaluation of *P. cookii* samples was performed by immunodetection of the BYDV coat protein in tissue prints on nitrocellulose membranes.

Briefly, stems were cut with a razor blade and firmly pressed onto the membrane (BA85, Schleicher & Schuell) for several seconds. The membrane was then treated as described by Fakhfakh et al. [24] with the following modifications: the saturation time was increased for 2 h and the membrane was incubated with a 1∶3,000 dilution of rabbit immunoglobulins raised against purified BYDV-PAV and BYDV-MAV virions (Adgen Phytodiagnostics, cat. no. 1030). It was then incubated to 2 h in the 1% gelatin radioimmunoassay buffer containing a 1∶5,000 dilution of rabbit immunoglobulins conjugated with alkaline phosphatase (Sigma, cat. no. A3812). Samples imprints were assessed visually with a binocular microscope at low magnification.

### Virus isolates and total nucleic acids extraction

All BYDV isolates included in this study are listed in Table S2. For RT-PCR analyses, Smart™ Long Distance (LD) PCR, and RACE PCR, total nucleic acids (TNA) were extracted from dried samples using the RNeasy Plant Minikit (Qiagen, cat. no. 74904) according to the manufacturer's instructions. RNA was resuspended in 40 µl of DNase- and RNase-free water and stored at −20°C until used.

### RT-PCR amplification assays

**Figure 1:**
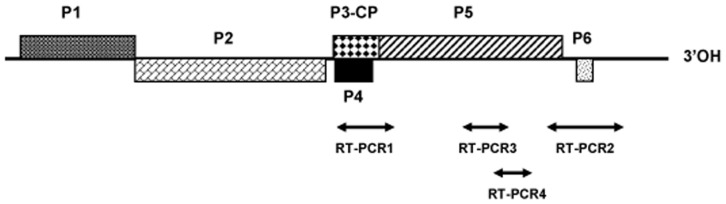
Genome organization of *Barley yellow dwarf virus* and position of regions amplified by RT-PCR assays.

Four RT-PCR assays with different specificities and targeting different parts of the BYDV genome were used in the present study. The position of the amplified regions on the BYDV genome is shown schematically on Fig. 1 while the sequence of the primers used and their annealing temperatures are given in supplementary Table S3.

The RT-PCR1 assay was used to detect viruses belonging to the *Luteovirus* genus, using polyvalent primers targeting the ORF3 region (primers Luteo1F and YanR-New, adapted from Malmstrom et al. [25]).

The RT-PCR2 assay uses the specific primer pair BYDV-P5-fw and BYDV-3′NC-rev2 designed on the reference isolate BYDV-PAV EF521849 sequence. This assay allows the amplification of a hypervariable 820-bp fragment overlapping the 3′ end of the ORF5 and the ORF6 of BYDV-PAV isolates.

The RT-PCR3 and RT-PCR4 assays targeting the ORF5 were designed to specifically amplify the divergent BYDV species (BYDV-Ch-fw1/BYDV-Ch-rev primer pair and ClusterB-spe-fw/ClusterB-spe-rev primer pair).

For all RT-PCR assays, a cDNA was synthesized from 5 µl of TNA initiated by N_6_ primers, using the Supercript®II Reverse Transcriptase according to the manufacturer's recommendations (Invitrogen, cat. no. 460624). In a second step, the PCR was carried out using 5 µl of cDNA for RT-PCR1 and 3 µl for RT-PCRs 2, 3 and 4 in a 50- µl reaction volume containing 10 mM Tris-HCl, pH 8.8, 1.5 mM MgCl_2_, 50 mM KCl, 0.1% Triton X-100, 250 µM dNTPs, 1 µM of each primer and 1 unit of DyNAzyme II™ DNA polymerase (Finnzymes, cat. no. F 501). After a denaturation step of 5 min at 95°C, forty amplification cycles were applied, each of 30 sec at 95°C, 30 sec at annealing temperature specific for each PCR (Table S3) and 1 min at 72°C for RT-PCRs 1 and 2, only 40 s for RT-PCRs 3 and 4. The 40 cycles were followed by a final extension step at 72°C during 10 min.

Amplification products were directly sequenced on both strands (Beckman Coulter Genomics). The sequences obtained were deposited in GenBank and accession numbers are listed in Table S2.

### Determination of the genome sequence of selected BYDV isolates

Double-stranded RNAs (dsRNAs) extracted from *P. cookii* infected by various BYDV isolates were analyzed by 454 pyrosequencing, as described by Candresse et al. [26], providing partial genomic sequences for isolates K439, K460 and K465. Specific primers designed from these partial sequences were then used to perform Long Distance PCR in order to validate the pyrosequencing data and to fill the gaps they contained. The specificity of the primers used as well as the annealing conditions are detailed in Table S3. The 5′ Race amplifications were performed as recommended by the kit supplier (Clontech, cat. no. 634923).

### Sequence analyses, comparisons and phylogenetic analyses

Multiple alignments of nucleotide or amino acid sequences were performed using the ClustalW program [27] as implemented in Mega 5.0 [28]. Pairwise strict nucleotide and amino acid distances were computed using Mega 5.0 and phylogenetic trees were reconstructed using the Neighbor joining method with bootstrap analysis in Mega 5.0. The RDP3 program was used to search for potential recombination events in the BYDV genomic sequences obtained during this work [29].

## Results

### Geographical distribution and hosts of BYDV in the Kerguelen Islands

Three hundred and seventy-eight *P. cookii* samples collected from 25 sampling sites (corresponding to 41 collection points, see Materials and Methods) were analyzed for the presence of BYDV by immunoprinting and/or by RT-PCR (Table 1). Ninety-four plants were found to be infected by BYDV, i.e a 24.9% prevalence. There was however a wide variability in infection rate between sites, the virus being detected in only 16 of the collection points (39%) and, when present, being observed with a prevalence ranging between 10.5% and 100%.

Since BYDV is known to be able to infect many members of the *Poaceae* family [16], the virus was also sought in various other grass species, with a higher sampling intensity in successive years in sites where high BYDV prevalence had been observed in *P. cookii*. In total, twenty one species of native or introduced grasses were tested by immunoprinting and/or RT-PCR for the presence of BYDV (Table 2). In addition to *P. cookii*, the virus was detected in two native species at one site each, *Festuca contracta* (Tufted Fescue, on the Ile Australia) and *Agrostis magellanica* (at the Port Couvreux site), and also in two introduced species, *Dactylis glomerata* (Cock's-foot, on the Ile aux Moules) and *Poa annua* (annual meadow grass or annual bluegrass, on the Ile aux Moules and the Ile Longue). In all these species, BYDV prevalence was lower than that observed in *P. cookii*, ranging from 4% (*A. magellanica*) to 7.7% (*F. contracta*).

Among the aphid species known to transmit BYDV [16], only *R. padi* has been recorded on the Kerguelen Islands. The other two aphid species known to occur in the wild are *Myzus ascalonicus* and *Myzus ornatus*, which are not known to transmit BYDV. As shown in Table 2, the plant species found to be infected by BYDV are among those observed to be the preferred hosts of *R. padi* [5], [6]. In addition, of the five grass species in which BYDV was detected, *P. annua* is the only biennial one, the other four being perennial.

**Figure 2:**
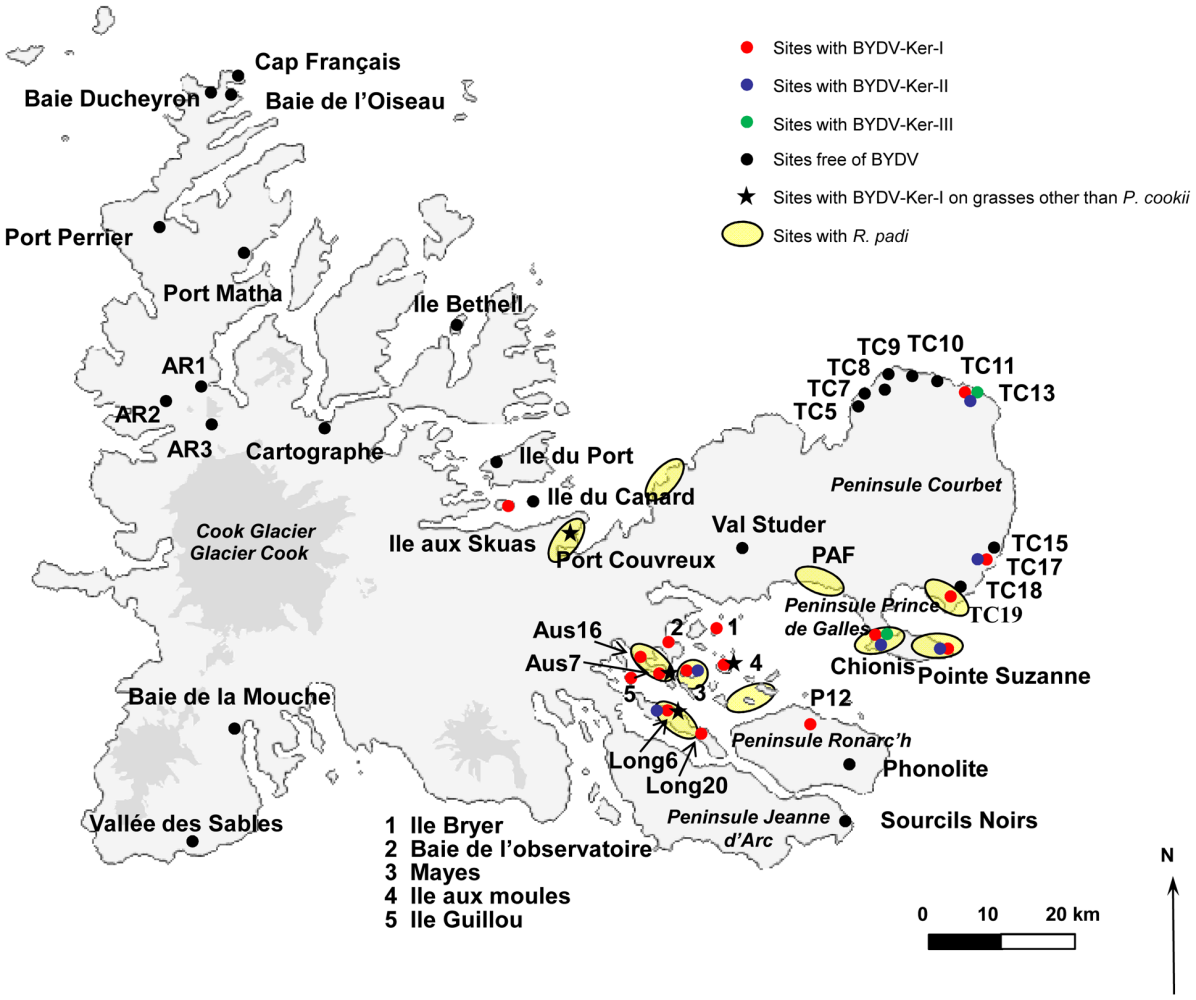
Geographical distribution of *Barley yellow dwarf virus* and *Rhopalosiphum padi* on the Kerguelen Islands.

Fig. 2 synthesizes the data on the geographical distribution of BYDV and *R. padi* on the Kerguelen Islands. The distribution of BYDV either on *P. cookii* or on other grasses appears to be limited to the south-east of the archipelago: West coast of the Péninsule Courbet, several islands in the Golfe du Morbihan (Ile Longue, Mayes, Australia), Presqu'île Ronarc'h, and Presqu'île du Prince de Galles (Chionis and Pointe Suzanne). On the other hand, the virus appears to be absent from the northern sampled sites. Due to their remoteness and difficulty of access, it has not been possible to sample sites on the west coast. Infection by BYDV on grasses other than *P. cookii* was also reported in four sites located in the Golfe du Morbihan (*P. annua* in Ile Longue, and Ile aux Moules, and *F. contracta* in Ile Australia) and in Port Couvreux (*A. magellanica*). As shown in Fig. 2, the distribution of BYDV shows good overlap with the known distribution of its aphid vector *R. padi*.

### Kerguelen Islands BYDV isolates comprise three major genetic lineages

**Figure 3:**
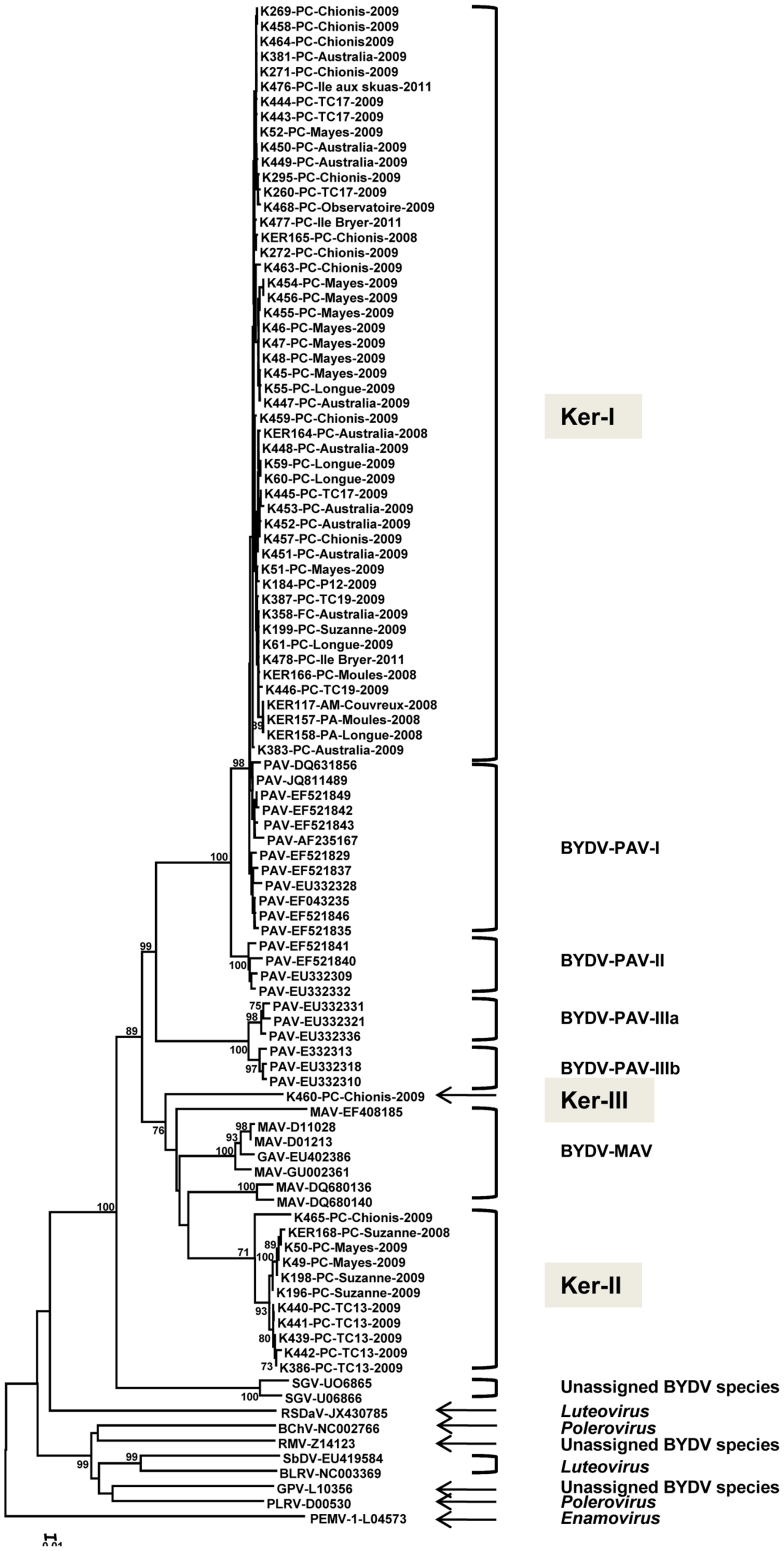
Phylogenetic tree reconstructed using the partial coat protein gene fragment amplified using polyvalent RT-PCR1 assay. The name of the isolate is followed by information on the host of origin, the sampling site and the year of collection. Hosts are given as follows: PC, *Poa cookii*, PA, *Poa annua*, FC, *Festuca contracta*, AM, *Agrostis magellanica*. Corresponding sequences from representative *Luteoviridae* members retrieved from GenBank were included in the analysis and the relevant accession numbers are indicated. The abbreviations used are: PAV: BYDV-PAV; MAV: BYDV-MAV; SGV: BYDV-SGV; RMV: BYDV-RMV; GPV: BYDV-GPV; RSDaV: *Rose spring dwarf associated virus*; BChV: *Beet chlorosis virus*; SbDV: *Soybean dwarf virus*; BLRV: *Bean leaf roll virus*; PLRV: *Potato leaf roll virus*; PEMV: *Pea enation mosaic virus*. The tree was reconstructed by using the neighbor-joining method, using the p-distance model and was bootstrapped with 1,000 replications. Numbers at nodes indicate bootstrap values higher than 70%. The scale bar represents 5% nucleotide divergence.

In order to analyse BYDV diversity, total nucleic acids extracts were submitted to the RT-PCR1 amplification assay. (Fig. 1 and Table S3). In total, the 500 nucleotide-long sequence was determined by direct sequencing of the PCR products obtained from 62 BYDV-isolates (Table S2). The corresponding sequences could be retrieved for 40 GenBank accessions corresponding to members of the *Luteoviridae* family and were included in the analysis. The phylogenetic tree in Fig. 3 shows the segregation of BYDV sequences from the Kerguelen Islands into three major lineages in the *Luteovirus* clade, which comprises BYDV-GAV, BYDV-PAV and BYDV-MAV and the tentative species BYDV-SGV. Each Kerguelen Islands lineage is supported by high bootstrap values (84 to 99%). The Ker-I lineage which comprises the majority of isolates (50/62, 80.6%) belongs to a very tight cluster corresponding to BYDV subspecies PAV-I [20]. The average pairwise nucleotide divergence (diversity) within this Ker-I cluster is only 0.6% (Table 3) while their average divergence with other members of the PAV-I cluster is 2.2% in the sequenced region. The Ker-II cluster regroups 11 sequences and is supported by a 99% bootstrap value (Fig. 3). It has a 1.7% average nucleotide diversity but exclusion of the K465 divergent isolate reduces this value to 0.8% for the 10 remaining Ker-II isolates (Table 3). Its closest affinities are with BYDV-MAV but with an average divergence of 17.9%, to be compared with the 24.8–25.1% divergence with the various BYDV-PAV clusters (Table 3). Lastly, a single isolate, K460 is isolated, with no close affinities, and constitutes the Ker-III cluster. This isolate shows 22.5%–28.4% divergence with the other BYDV clusters (Table 3).

**Table 3:** Average nucleotide divergence within and between *Barley yellow dwarf virus* clades for the partial CP gene region amplified using the Luteo1F and YanR-new primer pair.

	Ker-I	Ker-II	Ker-III	PAV-I	PAV-II	PAV-IIIa	PAV-IIIb	MAV
**Ker-I**	0.6							
**Ker-II**	25.0	1.7 (0.8^a^)						
**Ker-III**	25.6	22.5	na					
**PAV-I**	2.2	24.9	26.5	2.5				
**PAV-II**	5.4	25.1	22.5	5.1	1.5			
**PAV-IIIa**	21.2	25.1	28.4	21.5	21.3	1.2		
**PAV-IIIb**	21.0	24.8	28.0	21.4	21.1	3.8	1.1	
**MAV**	23.9	17.9	22.7	24.1	24.0	25.5	25.1	13.7

a calculated without K465 nucleotide sequence.

### Genetic diversity of BYDV-PAV-I isolates from Kerguelen Islands

**Figure 4:**
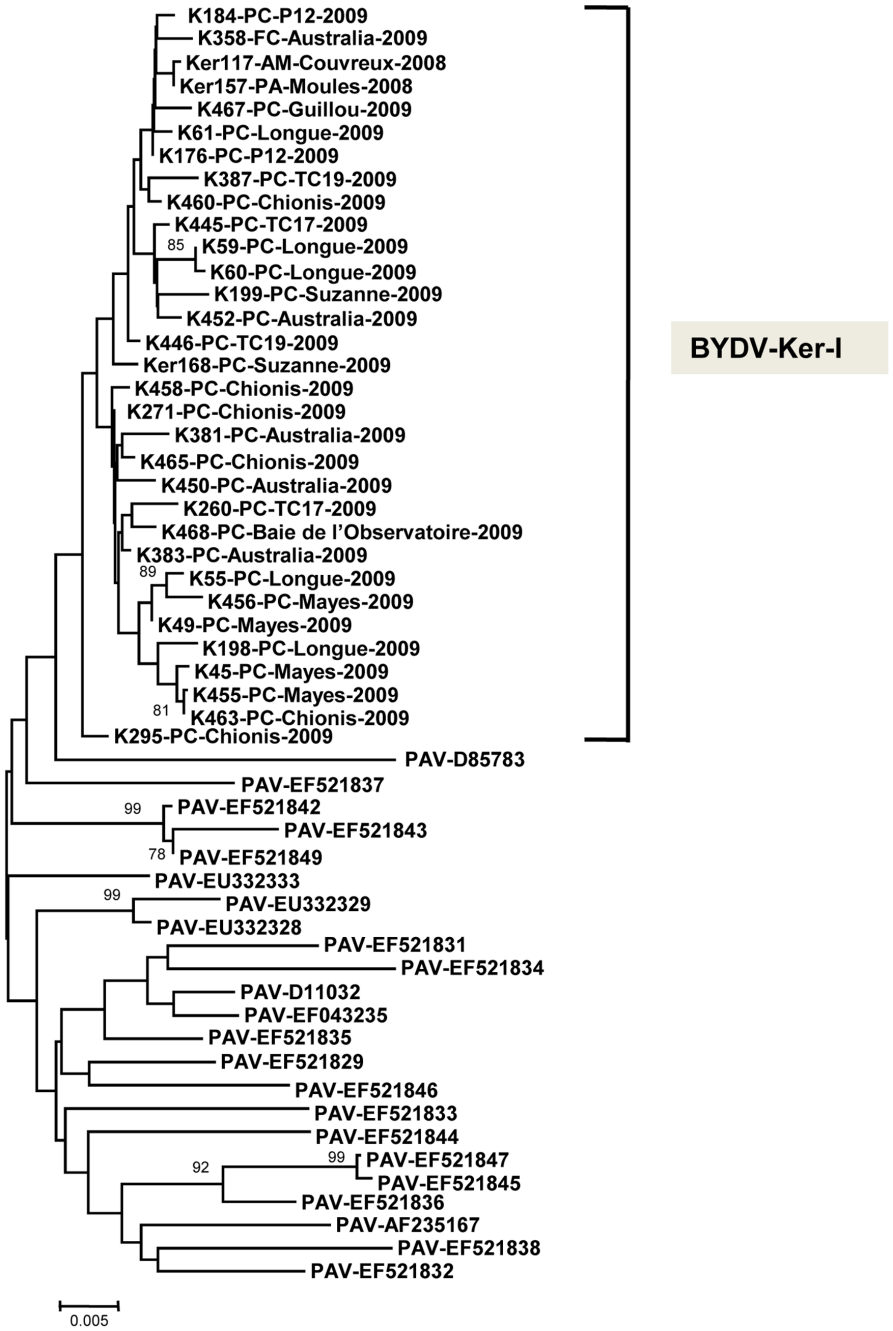
Unrooted phylogenetic tree reconstructed using the hypervariable sequence amplified on BYDV-Ker-I isolates using RT-PCR2 assay. Corresponding sequences from all BYDV-PAV isolates present in GenBank were included in the analysis and the relevant accession numbers are indicated. For Kerguelen isolates, the same additional information is provided as in Fig. 3. The tree was reconstructed by neighbor-joining using the p-distance model and was bootstrapped with 1,000 replications. Numbers at nodes indicate bootstrap values higher than 70%. The scale bar represents 0.5% nucleotide divergence.

The region amplified using the RT-PCR1 polyvalent assay shows high conservation within each of the BYDV-PAV subclusters and therefore does not allow the analysis of within-cluster relationships. A selected subset of Ker-I isolates was therefore amplified using the RT-PCR2 assay (Fig. 1 and Table S3) which amplifies an hypervariable 820-bp fragment corresponding to part of the gene encoding the P5 read-through aphid transmission protein (230 C-terminal amino acids) and to the gene coding for the highly variable P6 protein. This sequence was obtained for a total of 32 Ker-I isolates (Table S2) and compared to those of the 23 closest corresponding sequences in GenBank, which all belong to the PAV-I subcluster [20]. The analysis of the neighbor joining unrooted tree reconstructed (Fig. 4) shows that all the BYDV-Ker-I isolates cluster together, although the bootstrap value of the branch is not very high (44). In this hypervariable region, these isolates show an average diversity similar to that observed in the CP region (0.9%), while the diversity of the BYDV-PAV-I reference isolates included in this analysis, rises to 2.6% (not shown). This comparison indicates that the Ker-I isolates form an homogenous group of very low diversity, as expected for isolates deriving from a recent introduction event followed by a colonization phase.

### Molecular characterization of BYDV-Ker-II and BYDV-Ker-III isolates

The Ker-II and Ker-III clusters occupy isolated positions in the phylogenetic tree shown on Fig. 3, suggesting that they might represent distinct species or strains in the BYDV species complex. However, more sequence information is clearly needed before any firm conclusions can be drawn. The molecular characterization of isolates K439 (cluster Ker-II) and K460 (cluster Ker-III) was therefore undertaken. Additional sequencing was also performed on the divergent cluster Ker-II isolate K465.

Complete genomic sequences were determined for isolates K465 (5,763 nt, the 26 last nucleotides at the extreme 3′ end are those of the amplification primer) and K439 (5,736 nt, 14 nt at the 5′ end and 19 nt at the 3′ end are those of the amplification primers). The genome sizes are close to those determined for other BYDV isolates (between 5,647 and 5,706 nt). For the K460 isolate, about 80% of the genome sequence, from the partial P1 gene (lacking the N-terminal 13 or 14 amino acids) to the P5 one was determined (4,625 nt, the last 19 nt are those of the amplification primer). The three sequences show a typical *Luteovirus* genomic organization, with six ORFs encoded from 5′ to 3′ and corresponding respectively to the replication related proteins (ORF1 and ORF2), to a capsid protein (CP, ORF3) followed in frame by ORF5 encoding a protein thought to be implicated in the aphid transmission of the virus. The ORF4 probably involved in the viral long distance movement is located, as expected for luteoviruses, within ORF3. Finally, the small ORF6 is found near the 3′ end of the genome (no information for isolate K460 for which this region was not sequenced). When available, the 5′ and 3′ non-coding regions (NCR) are of similar size as those of BYDV-PAV. Comparisons of Ker-II cluster (K465 and K439 isolates) and representative members of BYDV species showed overall nucleotide identity levels comprised between 62.9 and 63.8%. The corresponding values obtained for the partial Ker-III isolate (K460) are between 65.6 and 66.5%. By comparison, less than 51% of overall nucleotide identity was observed with other members of the *Luteoviridae* family, confirming that Ker-II and Ker-III isolates belong to the BYDV species complex (Table S4).

**Figure 5:**
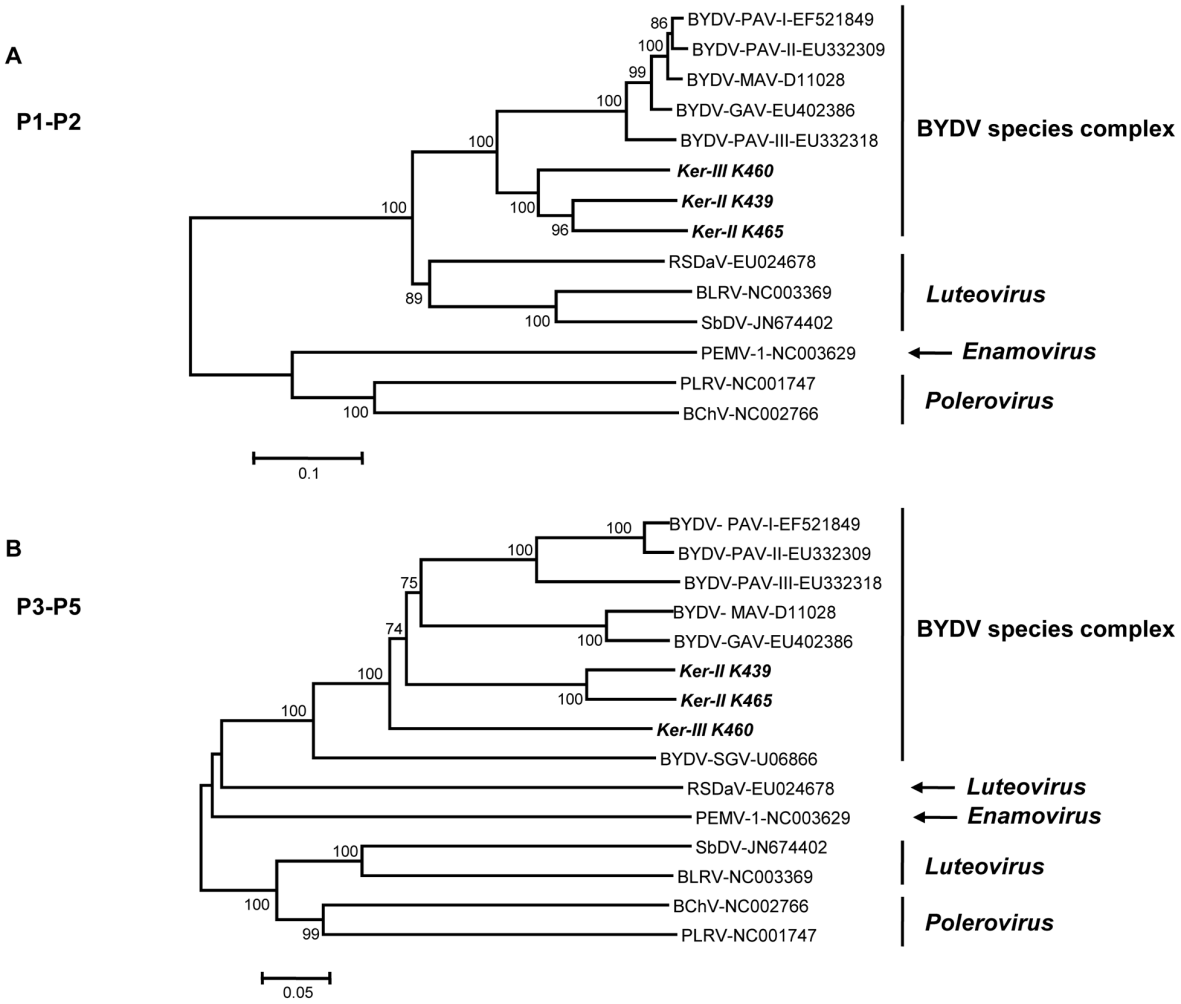
Phylogenetic trees reconstructed using the amino acid sequences for (A) the P1-P2 fusion protein and (B) the P3-P5 read-through protein of representative members of the family *Luteoviridae*. Trees were constructed by the neighbor-joining method and the statistical significance of branches was evaluated by bootstrap analysis (1,000 replicates). Only bootstrap values above 70% are indicated. The scale bars represent 10% (A) or 5% (B) amino acid divergence. BYDV-Ker-III K460, BYDV-Ker-II K465 and BYDV-Ker-II K439 are indicated in bold italics. The genera to which the particular viruses belong are indicated on the right. The abbreviations followed by the accession numbers are: RSDaV, *Rose spring dwarf-associated virus*; BLRV, *Bean leafroll virus*; SbDV, *Soybean dwarf virus*; PEMV, *Pea enation virus*; PLRV, *Potato leafroll virus*; BChV, *Beet chlorosis virus*.

Further confirmation is provided by the neighbor joining phylogenetic trees shown in Fig. 5, which were reconstructed using amino acid sequence identity distances for the P1-P2 fusion protein (Fig. 5A) and for the P3-P5 read-through protein (Fig. 5B). In each case, and also for the tree reconstructed with the entire genome sequences, the Ker-III (K460) and Ker-II isolates (K439 and K465) clustered together with other members of the BYDV species complex, with 100% bootstrap support. Comparisons between the amino acid sequences of the ORFs of Ker-II isolates and Ker-III with those of *Luteoviridae* members were performed (Tables S5, S6 and S7) and showed that whatever the ORF considered, at least 23%, 24% and 32% of amino acid divergence was observed for the K439, K465 and K460 isolates, respectively. The divergence is similarly high between the Ker-II and Ker-III isolates (at least 22% in the P1-P2 and Table S4). These comparisons in conjunction with the phylogenetic analysis strongly suggest that the Ker-II and Ker-III clusters represent two novel BYDV species.

The pattern of divergence between the two Ker-II isolates (K439 and divergent K465) is unusual, showing higher divergence in the 5′ genome half (in particular 34.3% amino acid divergence in the P1 protein, Table S6) and comparatively lower divergence levels for the 3′ genome part (5.1% divergence in the CP, overall 13% divergence for the complete P3-P5 read-through protein). Such a pattern suggests that as for other members of the BYDV species complex, some Ker-II isolates might result from recombination events [18]. A search for potential recombination events involving these two isolates was therefore performed using a dataset consisting of representative full length sequences available to each BYDV strain or species and the RDP3 program [29]. The results obtained suggest the existence of a recombination event involving the 5′ genome end of isolates K439 (typical Ker-II representative) and isolate K460 (Ker-III), up to around position 673 of the K439 sequence, which corresponds to near middle of ORF1 (probability for the absence of recombination evaluated between 10^−15^ to 10^−37^, depending on the particular program considered within RDP3, results not shown). The identity between the Ker-II and Ker-III isolates in that region is however still important, reaching 71.7 to 78% in P1 and P1-P2 ORFs, respectively (Tables S5 and S7), suggesting that the potential recombination event thus detected is an ancient one, since a much higher percentage of identity would have been expected in case of a recent recombination event.

### BYDV-Ker-II and BYDV-Ker-III diversity and evidence for mixed infections

Based on the genomic sequences of the K439 and K465 (BYDV-Ker-II) and K460 (BYDV-Ker-III) isolates, primers allowing the specific amplification of members of each cluster were designed, targeting the variable part of the P5 read-through aphid transmission protein gene (RT-PCRs 3 and 4, Table S3 and Fig. 1). A total of 94 *P. cookii* samples collected in sites where BYDV had been detected were specifically tested for the presence of isolates of these clusters. Table 4 summarizes the infection status data for the 17 samples in which isolates belonging to the Ker-II or Ker-III clusters were identified. Six plants were found to be infected by BYDV-Ker-III, and 16 by BYDV-Ker-II. Mixed infection situations involving isolates belonging to different clusters were also found, since 14 plants contained isolates from more than a single cluster (Table 4). In fact, isolates of the Ker-III cluster were not observed in single infection. The three possible double infections (Ker-I/Ker-II: eight plants, Ker-I/Ker-III: one plant and Ker-II/Ker-III: two plants) were observed, as was the triple infection (three plants).

**Table 4:** *Barley yellow dwarf virus* infection status of samples identified as containing either BYDV-Ker-II or BYDV-Ker-III isolates.

	RT-PCR1 (Ker-I, Ker-II, Ker-III)	RT-PCR2 (Ker-I)	RT-PCR3 (Ker-III)	RT-PCR4 (Ker-II)
**KER168-PC-Suzanne-2008**	Ker-II	Ker-I	−	Ker-II
**K198-PC-Suzanne-2009**	Ker-II	Ker-I	−	+
**K196-PC-Suzanne-2009**	Ker-I	nt	−	Ker-II
**K197-PC-Suzanne-2009**	+	+	−	+
**K50-PC-Mayes-2009**	Ker-II	−	−	Ker-II
**K49-PC-Mayes-2009**	Ker-II	Ker-I	−	Ker-II
**K460-PC-Chionis-2009**	Ker-III	Ker-I	Ker-III	−
**K461-PC-Chionis-2009**	Ker-I	nt	Ker-III	Ker-II
**K463-PC-Chionis-2009**	Ker-I	Ker-I	Ker-III	Ker-II
**K465-PC-Chionis-2009**	Ker-II	Ker-I	−	Ker-II
**K439-PC-TC13-2009**	Ker-II	+	−	Ker-II
**K440-PC-TC13-2009**	Ker-II	+	Ker-III	Ker-II
**K441-PC-TC13-2009**	Ker-II	+	Ker-III	Ker-II
**K442-PC-TC13-2009**	Ker-II	+	+	Ker-II
**K386-PC-TC13-2009**	Ker-II	−	−	Ker-II
**K445-PC-TC17-2009**	Ker-I	Ker-I	−	Ker-II
**K57-PC-Longue-2009**	+	−	−	Ker-II

The specificity of the various RT-PCR assays used is given in parentheses. The identity of the RT-PCR products was determined (polyvalent RT-PCR1) or confirmed (RT-PCR 2, 3 and 4) by direct sequencing of the amplified products. Positive amplifications for which identity of the product was not confirmed by sequencing are identified by a +. Negative amplification results are identified by a - sign. nt: not tested.

The distribution of BYDV-Ker-III seems to be more limited, because it was detected only in two sites (Chionis and TC13, Fig. 2) and the isolates involved show a very low diversity in the sequenced fragment (0.5% average nucleotide divergence). BYDV-Ker-II has a broader distribution because it was detected in six sites, to be compared with the 16 sites in which BYDV-Ker-I was detected (Fig. 2). Its nucleotide diversity was similar to that of Ker-III and Ker-I clusters (0.7% average pairwise divergence) when excluding the isolate K465. Indeed, in the sequenced region, isolate K465 is more divergent from other Ker-II isolates, with more than 20% of nucleotide divergence (23% divergence in the encoded amino acid sequence).

## Discussion

In this study, three BYDV species have been detected in the Kerguelen Islands: BYDV-PAV-I (Ker-I cluster), found to infect native and introduced *Poaceae* plants and the novel BYDV-Ker-II and BYDV-Ker-III species, detected with a lower prevalence and only in *P. cookii*. These two new species in the BYDV species complex clearly show genetic distance values with other known *Luteoviridae* outside the species demarcation criteria for the family (>10% differences at the amino acid level for any viral gene product) [17]. The molecular characterization of K465 and K439, two isolates of the Ker-II cluster, suggests that at least one recombination event, likely involving also the Ker-III cluster may have contributed to the evolutionary history of the Ker-II cluster. Some recent studies suggest that this situation is not rare in the BYDV complex, because BYDV-PAV exhibits a relatively high frequency of recombinant isolates [18], [19].

The discovery of BYDV in the Kerguelen Islands is surprising given that the aphid fauna is extremely limited and of relatively recent introduction, during the second half of the 20^th^ century (Voisin, unpublished data, and [7]). It is difficult to envision the widespread presence of BYDV in an aphid-free environment, given that this virus is not known to be mechanically or seed transmissible [16]. The most likely scenario is therefore that BYDV was introduced in the Kerguelen environment with the same recent time frame as its aphid vectors. Long distance spread of BYDV through maritime trade between Australia and the USA has previously been proposed on the basis of analysis of herbaria-preserved grasses samples [30]. Although the second most isolated archipelago on earth, the Kerguelen Islands were visited by whalers in the past and regular, if limited, boat traffic has existed for the second part of the 20^th^ century, if only to provide supplies and food to the limited but permanent human settlement mostly associated with scientific research [31]. In this context, introduction of BYDV on plants or by viruliferous aphids travelling on plant products seems to be the most likely scenario. The very low diversities observed for all three BYDV clusters are in good agreement with a recent introduction scenario, which would have likely imposed a strong genetic bottleneck on the introduced BYDV populations. The evolutionary rate of BYDV-PAV has been estimated at 3.158×10^−4^ substitutions/site/year [18]. Given the 0.5% to 0.9% diversity observed in the various regions analyzed for the three identified BYDV clusters, this would translate in a 16 to 28 years period since introduction, in good agreement with a recent introduction scenario. The absence of BYDV in some of the sampling sites, in particular in the north sites that are the most distant ones from the likely site of introduction, the permanent station established in 1950 at Port-Aux-Français [2], is also in good agreement with a recent, on-going invasion process in which the virus has not yet colonized the full extent of its available range. Moreover, the distribution of BYDV fits very well with that of its aphid vector, *R. padi* (Fig. 2). Nevertheless, in some collection points, the aphid was not observed (for example TC13, Table 1), although BYDV was detected in *P. cookii*. This could be explained either by a low, undetected aphid presence or, alternatively, by a past contamination event followed by local aphid extinction, since once established BYDV infection would be expected to locally persist in the perennial *P. cookii*. Variability in the abundance of *R. padi* population was indeed observed in Chionis site for example (Table 1).

The strongest counter argument against this recent introduction scenario is that two of the BYDV species identified (BYDV-Ker-II and BYDV-Ker-III) have no known counterpart elsewhere in the world. However, our knowledge of BYDV is still limited and novel BYDV species or subspecies have been described recently [20]. The most frequent ship contacts link the Kerguelen Islands with the Reunion Island in the Indian ocean, which itself has significant connections with the Mascareignes archipelago and eastern Africa, all regions for which we have essentially no information on local BYDV diversity.

An interesting question concerns the high prevalence of BYDV, irrespective of the cluster concerned, on *P. cookii* as compared to other native or introduced grass species. The perennial growth habit of *P. cookii* is unlikely to be the primary factor as BYDV was detected in several other perennial species, but with a 4 to 7-fold lower prevalence (Table 2). One hypothesis would be that *P. cookii* physiological phenotype may explain its greater reservoir potential [32] or that *P. cookii* could have an otherwise greater attractivity for the aphid vectors than other grass species. *R. padi* is the second most abundant and common aphid species in the Kerguelen Islands, after *M. ascalonicus*. A third species, *M. ornatus* is also recorded in the wild but with lower abundance [6]. Of these, *R. padi* is the only known vector of BYDV [33] and was detected in 25% of the sites prospected [6], [34], with quite good match between its distribution and that reported here for BYDV, in particular the absence of both vector and virus from the northern-most sampling sites. However, *R. padi* was observed to colonize and be locally abundant not only on *P. cookii* but also on other grass species, in particular *F. contracta, P. annua, A. geniculatus* and *H. lanatus* (Table 2) [6], [34]. There is therefore a poor correlation between the level of *R. padi* infestation and BYDV prevalence in the various grass species, suggesting that attractiveness to aphids is unlikely to explain the greater prevalence of BYDV in *P. cookii*.

There is currently no information on the aphid species able to transmit BYDV-Ker-II and BYDV-Ker-III. The fact that isolates belonging to cluster BYDV-Ker-II were observed in single infection (Table 4) suggests that they can be transmitted by *R. padi*, which is able to transmit a range of BYDV species with variable efficiency. No such conclusion can be reached for isolates of BYDV-Ker-III, since this less prevalent virus has not been observed in single infection (Table 4). Two hypotheses can therefore be envisioned, that BYDV-Ker-III isolates are directly transmissible by *R. padi* or that they are transmitted by this vector through heterologous encapsidation in BYDV-Ker-I (or BYDV-Ker-II) particles, as reported for other BYDV species [35], [36].

Whether the introduction of *R. padi* and of BYDV and the predicted increase in suitability of this environment for aphids as a consequence of global warming [5] will further endanger the fragile Kerguelen Islands ecosystem remains to be precisely evaluated.

## Supporting Information

Table S1 **GPS position of collection sites.**
(DOCX)

Table S2 **List of** ***Barley yellow dwarf virus*** **isolates included in the present study, indicating their original host, the year and site of collection and the GenBank accession numbers of the partial or complete sequences obtained.**
(DOCX)

Table S3 **Primers used in this study.**
(DOCX)

Table S4 **Percentage of overall genome nucleotide sequence identity between BYDV-Ker-III K460, BYDV-Ker-II K465 and BYDV-Ker-II K439 isolates and members of** ***Luteoviridae*** **family.**
(DOCX)

Table S5 **Percentage of amino acid divergence observed between the various genomic regions of BYDV-Ker-III K460 and members of** ***Luteoviridae*** **family.**
(DOCX)

Table S6 **Percentage of amino acid divergence observed between the various genomic regions of BYDV-Ker-II K465 and members of** ***Luteoviridae*** **family.**
(DOCX)

Table S7 **Percentage of amino acid divergence observed between the various genomic regions of BYDV-Ker-II K439 and members of** ***Luteoviridae*** **family.**
(DOCX)
