# Supplementary material for: Distribution of Barley yellow dwarf virus-PAV in the Sub-Antarctic Kerguelen Islands and Characterization of Two New Luteovirus Species
Source: PLoS One. 2013 Jun 18;8(6):e67231. doi: 10.1371/journal.pone.0067231 (PMC3688969; doi:10.1371/journal.pone.0067231)
Supplement: Table S1 — GPS position of collection sites. (DOCX) [file pone.0067231.s001.docx]

**Table S1.**GPS position of collection sites

| **Prospected sites** | **Collected Point** | **GPS position** |
| --- | --- | --- |
| Presqu’île Ronarc’h | P12 | S49 32.478 E70 09.717 |
|  |  | S49 33.065 E70 12.049 |
|  | Phonolite | S49 36.576 E70 15.768 |
| Ile Longue | Long6 | S49 31.417 E69 53.973 |
|  |  | S49 32.038 E69 53.421 |
|  |  | S49 32.090 E69 53.814 |
|  |  | S49 31.943 E69 55.397 |
|  | Long20 | S49 33.415 E69 57.149 |
|  |  | S49 33.882 E69 58.877 |
| Observatoire | Observatoire | S49 24 E69 53^a^ |
| Ile Guillou | Ile Guillou | S49 28.190 E69 48.470 |
| Ile Australia | Aus7 | S49 29.313 E69 54.046 |
|  |  | S49 28.901 E69 53.589 |
|  |  | S49 28.796 E69 53.472 |
|  |  | S49 28.482 E69 52.896 |
|  |  | S49 28.481 E69 52.897 |
|  |  | S49 28.741 E69 53.701 |
|  | Aus16 | S49 27.945 E69 52.548 |
|  |  | S49 28.016 E69 52.362 |
|  |  | S49 27.822 E69 51.772 |
|  |  | S49 27.638 E69 51.557 |
|  |  | S49 26.853 E69 49.956 |
|  |  | S49 28.265 E69 52.250 |
| Ile Mayes | Ile Mayes | S49 28.033 E69 55.762 |
|  |  | S49 28.039 E69 55.592 |
|  |  | S49 28.165E69 56.512 |
| Péninsule Courbet | TC5 | S49 05.842 E70 14.363 |
|  |  | S49 05.498 E70 14.585 |
|  | TC7 | S49 04.388 E70 16.874 |
|  | TC8 | S49 04.323 E70 18.948 |
|  | TC9 | S49 03.428 E70 20.741 |
|  | TC10 | S49 03.432 E70 23.076 |
|  | TC11 | S49 04.225 E70 25.704 |
|  |  | S49 03.986 E70 25.373 |
|  | TC13 | S49 04.749 E70 28.179 |
|  | TC15 | S49 17.539 E70 32.039 |
|  |  | S49 18.171 E70 31.977 |
|  | TC17 | S49 18.873 E70 31.279 |
|  | TC18 | S49 21.157 E70 28.963 |
|  | TC19 | S49 21.589 E70 27.633 |
| Presqu’île du Prince de Galles | Chionis | S49 26.108 E70 16.928 |
|  |  | S49 26.097 E70 16.947 |
|  |  | S49 26.077 E70 16.964 |
| Anse du Cartographe | Cartographe | S49 07 40 E69 12 59^a^ |
| Presqu’île Jeanne d’Arc | Sourcils noirs | S49 41 262 E70 14 616^a^ |
| Val Studer | Studer | S49 17.889 E70 02.344 |
| Ile du Port | Ile du Port | S49 11.030 E69 38.940 |
| Port Perrier | Port Perrier | S48 53.442 E68 54.810 |
|  |  | S48 53.203 E68 53.704 |
| Anse Ring | AR1 | S49 05.286 E68 59.197 |
|  |  | S49 05.381 E69 00.207 |
|  | AR2 | S49 06.574 E68 55.248 |
|  | AR3 | S49 09.049 E6901.203 |
| Ile aux Moules | Ile aux Moules | S49 24 E69 56^a^ |
| Ile du Canard | Ile du Canard | S4914.172E6936.553 |
| Ile aux Skuas | Ile aux Skuas | S4515.062E6934.218 |
| Ile Bryer | Ile Bryer | S4925.001E6958.538 |
| Baie de la Mouche | Baie de la Mouche | S4933.301E6903.045 |
| Vallée des Sables | Vallée des Sables | S4942.755E6855.862 |
| Cap Français | Cap Français | S48 40 15 E69 03 44^a^ |
| Baie de l’Oiseau | Baie de l’Oiseau | S48 40.556 E69 00.994 |
| Baie Ducheyron | Baie Ducheyron | S48 40.199 E69 00.589 |
| Port Matha | Port Matha | S48 56 E69 03^a^ |
| Ile Bethell | Ile Bethell | S4900851E6928185 |

^a^These positions are approximate
