# Supplementary material for: Distribution of Barley yellow dwarf virus-PAV in the Sub-Antarctic Kerguelen Islands and Characterization of Two New Luteovirus Species
Source: PLoS One. 2013 Jun 18;8(6):e67231. doi: 10.1371/journal.pone.0067231 (PMC3688969; doi:10.1371/journal.pone.0067231)
Supplement: Table S2 — List of Barley yellow dwarf virus isolates included in the present study, indicating their original host, the year and site of collection and the GenBank accession numbers of the partial or complete sequences obtained. (DOCX) [file pone.0067231.s002.docx]

**Table S2.** List of *Barley yellow dwarf virus* isolates included in the present study, indicating their original host, the year and site of collection and the GenBank accession numbers of the partial or complete sequences obtained

| **Virus** | **Sample** | **Year of collection** | **Collection site** | **Host** | **Accession numbers^a^** |
| --- | --- | --- | --- | --- | --- |
| BYDV-PAV-I | KER117 | 2008 | Port Couvreux | *Agrostis magellanica* | KC292609 - KC292523 |
| BYDV-PAV-I | KER157 | 2008 | Moules | *Poa annua* | KC292610 - KC292524 |
| BYDV-PAV-I | KER158 | 2008 | Ile Longue | *Poa cookii* | KC292525 |
| BYDV-PAV-I | KER164 | 2008 | Ile Australia | *Poa cookii* | KC292528 |
| BYDV-PAV-I | KER165 | 2008 | Chionis | *Poa cookii* | KC292527 |
| BYDV-PAV-I | KER168 | 2008 | Pointe Suzanne | *Poa cookii* | KC292612 |
| BYDV-PAV-I | KER166 | 2008 | Ile aux Moules | *Poa cookii* | KC292526 |
| BYDV-PAV-I | K45 | 2009 | Ile Mayes | *Poa cookii* | KC292582 - KC292550 |
| BYDV-PAV-I | K46 | 2009 | Ile Mayes | *Poa cookii* | KC292539 |
| BYDV-PAV-I | K47 | 2009 | Ile Mayes | *Poa cookii* | KC292551 |
| BYDV-PAV-I | K48 | 2009 | Ile Mayes | *Poa cookii* | KC292552 |
| BYDV-PAV-I | K49 | 2009 | Ile Mayes | *Poa cookii* | KC292605 |
| BYDV-PAV-I | K51 | 2009 | Ile Mayes | *Poa cookii* | KC292541 |
| BYDV-PAV-I | K52 | 2009 | Ile Mayes | *Poa cookii* | KC292540 |
| BYDV-PAV-I | K55 | 2009 | Ile Longue | *Poa cookii* | KC292592 - KC292542 |
| BYDV-PAV-I | K59 | 2009 | Ile Longue | *Poa cookii* | KC292583 - KC292544 |
| BYDV-PAV-I | K60 | 2009 | Ile Longue | *Poa cookii* | KC292593 - KC292549 |
| BYDV-PAV-I | K61 | 2009 | Ile Longue | *Poa cookii* | KC292584 - KC292543 |
| BYDV-PAV-I | K176 | 2009 | P12 | *Poa cookii* | KC292606 |
| BYDV-PAV-I | K184 | 2009 | P12 | *Poa cookii* | KC292588 - KC292545 |
| BYDV-PAV-I | K198 | 2009 | Ile Longue | *Poa cookii* | KC292613 |
| BYDV-PAV-I | K199 | 2009 | Pointe Suzanne | *Poa cookii* | KC292585 - KC292536 |
| BYDV-PAV-I | K260 | 2009 | TC17 | *Poa cookii* | KC292587 -KC292557 |
| BYDV-PAV-I | K269 | 2009 | Chionis | *Poa cookii* | KC292532 |
| BYDV-PAV-I | K271 | 2009 | Chionis | *Poa cookii* | KC292594 - KC292531 |
| BYDV-PAV-I | K272 | 2009 | Chionis | *Poa cookii* | KC292529 |
| BYDV-PAV-I | K295 | 2009 | Chionis | *Poa cookii* | KC292586 - KC292530 |
| BYDV-PAV-I | K358 | 2009 | Ile Australia | *Festuca contracta* | KC292608 - KC292547 |
| BYDV-PAV-I | K381 | 2009 | Ile Australia | *Poa cookii* | KC292595 - KC292548 |
| BYDV-PAV-I | K383 | 2009 | Ile Australia | *Poa cookii* | KC292596 - KC292546 |
| BYDV-PAV-I | K387 | 2009 | TC19 | *Poa cookii* | KC292590 - KC292561 |
| BYDV-PAV-I | K443 | 2009 | TC17 | *Poa cookii* | KC292558 |
| BYDV-PAV-I | K444 | 2009 | TC17 | *Poa cookii* | KC292559 |
| BYDV-PAV-I | K445 | 2009 | TC17 | *Poa cookii* | KC292589 - KC292560 |
| BYDV-PAV-I | K446 | 2009 | TC19 | *Poa cookii* | KC292591 - KC292562 |
| BYDV-PAV-I | K447 | 2009 | Ile Australia | *Poa cookii* | KC292563 |
| BYDV-PAV-I | K448 | 2009 | Ile Australia | *Poa cookii* | KC292564 |
| BYDV-PAV-I | K449 | 2009 | Ile Australia | *Poa cookii* | KC292565 |
| BYDV-PAV-I | K450 | 2009 | Ile Australia | *Poa cookii* | KC292597 - KC292566 |
| BYDV-PAV-I | K451 | 2009 | Ile Australia | *Poa cookii* | KC292567 |
| BYDV-PAV-I | K452 | 2009 | Ile Australia | *Poa cookii* | KC292598 - KC292568 |
| BYDV-PAV-I | K453 | 2009 | Ile Australia | *Poa cookii* | KC292569 |
| BYDV-PAV-I | K454 | 2009 | Ile Mayes | *Poa cookii* | KC292570 |
| BYDV-PAV-I | K455 | 2009 | Ile Mayes | *Poa cookii* | KC292599 - KC292571 |
| BYDV-PAV-I | K456 | 2009 | Ile Mayes | *Poa cookii* | KC292600 - KC292572 |
| BYDV-PAV-I | K457 | 2009 | Chionis | *Poa cookii* | KC292573 |
| BYDV-PAV-I | K458 | 2009 | Chionis | *Poa cookii* | KC292601 - KC292574 |
| BYDV-PAV-I | K459 | 2009 | Chionis | *Poa cookii* | KC292575 |
| BYDV-PAV-I | K460 | 2009 | Chionis | *Poa cookii* | KC292602 |
| BYDV-PAV-I | K463 | 2009 | Chionis | *Poa cookii* | KC292603 - KC292576 |
| BYDV-PAV-I | K464 | 2009 | Chionis | *Poa cookii* | KC292577 |
| BYDV-PAV-I | K465 | 2009 | Chionis | *Poa cookii* | KC292611 |
| BYDV-PAV-I | K467 | 2009 | Guillou | *Poa cookii* | KC292607 |
| BYDV-PAV-I | K468 | 2009 | Chionis | *Poa cookii* | KC292604 - KC292578 |
| BYDV-PAV-I | K476 | 2011 | Ile aux Skuas | *Poa cookii* | KC292579 |
| BYDV-PAV-I | K477 | 2011 | Ile Bryer | *Poa cookii* | KC292580 |
| BYDV-PAV-I | K478 | 2011 | Ile Bryer | *Poa cookii* | KC292581 |
| BYDV-Ker-II | KER168 | 2008 | Pointe Suzanne | *Poa cookii* | KC292619 - KC292533 |
| BYDV-Ker-II | K49 | 2009 | Mayes | *Poa cookii* | KC292620 - KC292537 |
| BYDV-Ker-II | K50 | 2009 | TC13 | *Poa cookii* | KC292621 - KC292538 |
| BYDV-Ker-II | K196 | 2009 | Pointe Suzanne | *Poa cookii* | KC292622 - KC292534 |
| BYDV-Ker-II | K198 | 2009 | Pointe Suzanne | *Poa cookii* | KC292535 |
| BYDV-Ker-II | K386 | 2009 | TC13 | *Poa cookii* | KC292618 - KC292554 |
| BYDV-Ker-II | K440 | 2009 | TC13 | *Poa cookii* | KC292623 - KC292555 |
| BYDV-Ker-II | K441 | 2009 | TC13 | *Poa cookii* | KC292624 - KC292556 |
| BYDV-Ker-II | K442 | 2009 | TC13 | *Poa cookii* | KC292625 -KC292553 |
| BYDV-Ker-II | K445 | 2009 | TC17 | *Poa cookii* | KC292626 |
| BYDV-Ker-II | K461 | 2009 | Chionis | *Poa cookii* | KC292628 |
| BYDV-Ker-II | K463 | 2009 | Chionis | *Poa cookii* | KC292627 |
| BYDV-Ker-II | K465 | 2009 | Chionis | *Poa cookii* | KC572000 |
| BYDV-Ker-II | K439 | 2009 | TC13 | *Poa cookii* | KC571999 |
| BYDV-Ker-III | K440 | 2009 | TC13 | *Poa cookii* | KC292614 |
| BYDV-Ker-III | K441 | 2011 | TC13 | *Poa cookii* | KC292615 |
| BYDV-Ker-III | K461 | 2009 | Chionis | *Poa cookii* | KC292616 |
| BYDV-Ker-III | K463 | 2009 | Chionis | *Poa cookii* | KC292617 |
| BYDV-Ker-III | K460 | 2009 | Chionis | *Poa cookii* | KC559092 |

^a^When more than one PCR product was obtained for a given viral isolate, the various accession numbers for each sequenced genomic region are provided.
