# Supplementary material for: Distribution of Barley yellow dwarf virus-PAV in the Sub-Antarctic Kerguelen Islands and Characterization of Two New Luteovirus Species
Source: PLoS One. 2013 Jun 18;8(6):e67231. doi: 10.1371/journal.pone.0067231 (PMC3688969; doi:10.1371/journal.pone.0067231)
Supplement: Table S3 — Primers used in this study. (DOCX) [file pone.0067231.s003.docx]

**Table S3.** Primers used in this study.

|  | **Primer name** | **Primer sequence (5’>3’)** | **5’ position** | **Specificity** | **Annealing T°** |
| --- | --- | --- | --- | --- | --- |
| **RT-PCR1** | Luteo1F | ttcggmsartggttgtggtcca | PAV-I EF521849: 2931 | BYDV-*Luteovirus* | 56°C |
|  |  |  | MAV D11028: 2892 |  |  |
|  |  |  | GAV EU402386: 2926 |  |  |
|  | YanR-new^a^ | tgttgaggagtctacctatttng | PAV-I EF521849: 3475 |  |  |
|  |  |  | MAV D11028: 3436 |  |  |
|  |  |  | GAV EU402386: 3470 |  |  |
| **RT-PCR2** | BYDV-P5-fw | acttggaacataccagggacag | PAV-I EF521849: 4516 | BYDV-PAV | 64°C |
|  | BYDV-3’NC-rev2 | gtcttcaatcctgacgatcgg | PAV-I EF521849: 5336 |  |  |
| **RT-PCR3** | BYDV-Ch-fw1 | aggctccatgcttagacac | K460 : 4026 | BYDV-Ker-III | 56°C |
|  | BYDV-Ch-rev | cctgcgtcagctgttctac | K460 : 4451 |  |  |
| **RT-PCR4** | ClusterB-spe-fw | gatatcactagctctgagcg | K439: 4271 | BYDV-Ker-II | 58°C |
|  | ClusterB-spe-rev | ctctaagcctctcagccac | K439: 4634 |  |  |
| **K460 genome** | K460-fw3-all | ATWGGAGCTAGYRYYAARGC | K460: -20 | BYDV-Ker-III | 60°C |
|  | K460-rev2 | TTTGTTCACGACAGCTTGGG | K460: 1284 |  |  |
|  | K460-fw2-all | GARATYKCGGTCACAGAYGG | K460: 1039 | BYDV-Ker-III | 54°C |
|  | K460-rev1 | GAATTTCTTTTCATCCTTCC | K460: 1992 |  |  |
|  | PMGAV-GDD-fw | GGAGACGACTGTGTCATC | K460: 1945 | BYDV-Ker-III | 56°C |
|  | PMGAV-rev1-LD2 | tgtgtgcacgaagtgtcgag | K460: 3080 |  |  |
|  | PMGAV-rev1 in conjonction with Luteo1F | ctgcctgtttcccaggatccg | K460: 4626 | BYDV-Ker-III | 64°C |
| **K439 genome** | 5’-UPM | AGTGAAGATTGACC | K439: 1 | BYDV-Ker-II | Providing by the kit supplier |
|  | K439-5'Race3 | TAGTCCTCTGGTACAGGTATGCTCCCAG | K439: 507 |  |  |
|  | K439-LD5-fw | GGAGCTAGCGTCAAAGCC | K439: 179 | BYDV-Ker-II | 64°C |
|  | K439-5’Race | GGCCGAACTGTTCTATTGCTCCTGGCTC | K439: 2939 |  |  |
|  | Ker-MAV-pol-fw | ACCCACCCGCTACACAATAG | K439: 2738 | BYDV-Ker-II | 60°C |
|  | Ker-MAV-cont5-R | ACGTGCCTAAGCACTGCCTG | K439: 4955 |  |  |
|  | K439-LD3-fw | agtatggtctcaccgctgc | K439: 4826 | BYDV-Ker-II | 58°C |
|  | K439-LD3-rev | gtaaggtggacacaagagc | K439: 5736 |  |  |
| **K465 genome** | K465-5'Race1 | GCCATGGCTAGCTCTTCTTCTGACGCAAC | K465: 399 | BYDV-Ker-II | Providing by the supplier |
|  | K460-fw3-all | ATWGGAGCTAGYRYYAARGCCC | K465: 176 | BYDV-Ker-II | 60°C |
|  | ClusterBb-5'Race | ATCAGGCAGGCGCACTTCGTGAGG | K465: 1067 |  |  |
|  | K465-fw1 | GCTCGTCAACAACGAGGAGATAC | K465: 817 | BYDV-Ker-II | 56°C |
|  | K465-rev2 | AGGCCAATTGCAGAATTGGATTG | K465: 1948 |  |  |
|  | K465-fw2 | TTGAGTCCCTCAAAGTTCG | K465: 1556 | BYDV-Ker-II | 56°C |
|  | K465-rev3 | AAGCTTGAGGTCCTTGTGA | K465: 3945 |  |  |
|  | K465-fw3 | ACCAAAAGCACCCATGAGATCTC | K465: 3525 | BYDV-Ker-II |  |
|  | K465-rev1 | CTGTCAAAGTGCCGGACCCTTTC | K465: 5763 |  |  |

M: A or C; S: C or G; N: A, C, T or G; Y: C or T; R: A or G; K: G or T; W: A or T

^a^Adapted from [25]
