# Supplementary material for: Distribution of Barley yellow dwarf virus-PAV in the Sub-Antarctic Kerguelen Islands and Characterization of Two New Luteovirus Species
Source: PLoS One. 2013 Jun 18;8(6):e67231. doi: 10.1371/journal.pone.0067231 (PMC3688969; doi:10.1371/journal.pone.0067231)
Supplement: Table S4 — Percentage of overall genome nucleotide sequence identity between BYDV-Ker-III K460, BYDV-Ker-II K465 and BYDV-Ker-II K439 isolates and members of Luteoviridae family. (DOCX) [file pone.0067231.s004.docx]

**Table S4.** Percentage of overall genome nucleotide sequence identity between BYDV-Ker-III K460, BYDV-Ker-II K465 and BYDV-Ker-II K439 isolates and members of *Luteoviridae* family.

|  | BYDV PAV-I EF521849 | BYDV PAV-II EU332309 | BYDV PAV-III EU332318 | BYDV MAV D11028 | BYDV GAV EU402386 | BYDV Ker-III K460 | BYDV Ker-II K439 | BYDV Ker-II K465 | BLRV NC003369 | RSDaV EU024678 | SbDV JN674402 | PEMV-1 NC003629 | BChV NC002766 |
| --- | --- | --- | --- | --- | --- | --- | --- | --- | --- | --- | --- | --- | --- |
| BYDV PAV-I EF521849 |  |  |  |  |  |  |  |  |  |  |  |  |  |
| BYDV PAV-II EU332309 | 93.1 |  |  |  |  |  |  |  |  |  |  |  |  |
| BYDV PAV-III EU332318 | 81.3 | 81.5 |  |  |  |  |  |  |  |  |  |  |  |
| BYDV MAV D11028 | 82.8 | 82.1 | 77.5 |  |  |  |  |  |  |  |  |  |  |
| BYDV GAV EU402386 | 80.9 | 81 | 79 | 90.4 |  |  |  |  |  |  |  |  |  |
| BYDV Ker-III K460 KC559092 | 66.5 | 66.1 | 65.6 | 66.2 | 65.7 |  |  |  |  |  |  |  |  |
| BYDV Ker-II K439 KC571999 | 62.8 | 62.6 | 62.9 | 63.8 | 63 | 68 |  |  |  |  |  |  |  |
| BYDV Ker-II K465 KC572000 | 62.9 | 62.4 | 62.6 | 63.3 | 62.5 | 66.4 | 85 |  |  |  |  |  |  |
| BLRV NC003369 | 48.3 | 49 | 48 | 49.5 | 48.5 | 50.1 | 47.9 | 47.6 |  |  |  |  |  |
| RSDaV EU024678 | 51.2 | 51.5 | 50.9 | 51.4 | 50.7 | 52.3 | 50.5 | 50 | 48.2 |  |  |  |  |
| SbDV JN674402 | 48.1 | 49 | 47.7 | 48.5 | 47.9 | 49.7 | 47.5 | 46.8 | 65.6 | 47.2 |  |  |  |
| PEMV-1 NC003629 | 38.6 | 38.6 | 38.2 | 38.7 | 38.4 | 37.5 | 37 | 36.9 | 39.8 | 35.6 | 39.6 |  |  |
| BChV NC002766 | 37.8 | 37.7 | 37.4 | 37 | 37.4 | 38.2 | 37.4 | 37.5 | 38.2 | 36.4 | 38.6 | 55.3 |  |
| PLRV NC001747 | 35.4 | 35.6 | 36.1 | 35.1 | 35.5 | 35.8 | 35.4 | 35.1 | 35.2 | 34.4 | 34.3 | 41.3 | 41.6 |
