# Supplementary material for: Distribution of Barley yellow dwarf virus-PAV in the Sub-Antarctic Kerguelen Islands and Characterization of Two New Luteovirus Species
Source: PLoS One. 2013 Jun 18;8(6):e67231. doi: 10.1371/journal.pone.0067231 (PMC3688969; doi:10.1371/journal.pone.0067231)
Supplement: Table S5 — Percentage of amino acid divergence observed between the various genomic regions of BYDV-Ker-III K460 and members of Luteoviridae family. (DOCX) [file pone.0067231.s005.docx]

**Table S5.** Percentage of amino acid divergence observed between the various genomic regions of BYDV-Ker-III K460 and members of *Luteoviridae* family

| **Virus name and accession number** | **P1 (N-ter partial 327 aa)** | **P1-P2 (N-ter partial 856 aa)** | **P3 (200 aa)** | **P3-P5 (626 aa)** | **P4 (156 aa)** |
| --- | --- | --- | --- | --- | --- |
| BYDV-PAV-I EF521849 | 48.0 | 32.0 | 40.6 | 40.4 | 35.9 |
| BYDV-PAV-II EU332309 | 49.2 | 32.7 | 40.1 | 40.5 | 35.3 |
| BYDV-PAV-III EU332318 | 48.6 | 32.5 | 43.4 | 40.2 | 37.3 |
| BYDV-MAV D11028 | 48.9 | 32.7 | 34.2 | 40.6 | 31.8 |
| BYDV-GAV EU402386 | 48.0 | 32.6 | 34.7 | 41.4 | 31.2 |
| BYDV-GPV L10356 | na^c^ | na ^c^ | 58.5 | na ^c^ | 72.9 |
| BYDV-SGV U06866 | na ^c^ | na ^c^ | 40.8 | 49.8 | 41.1 |
| BYDV-RMV Z14123 | na ^c^ | na ^c^ | 57.0 | na ^c^ | 73.8 |
| BYDV-Ker-II K465 KC572000 | 42.8 | 28.4 | 34.7 | 40.1 | 32.2 |
| BYDV-Ker-II K439 KC571999 | 28.3 | 22.0 | 34.7 | 39.2 | 32.9 |
| BLRV NC003369 | 70.9 | 50.0 | 63.0 | 67.5 | 71.6 |
| RSDaV EU024678 | 61.9 | 47.0 | 63.3 | 70.1 | 74.8 |
| SbDV JN674402 | 70.0 | 49.2 | 61.7 | 66.2 | 71.2 |
| PEMV-1 NC003629 | 85.6 | 91.5 | 71.7 | 69.1 | na ^c^ |
| BChV NC002766 | 89.6 | 89.7 | 59.9 | 68.3 | 74.2 |
| PLRV NC001747 | 93.2 | 90.8 | 61.8 | 68.4 | 70.1 |

The P6 region, the 5’ non coding region and the 3’non coding region were not determined for BYDV-Ker-III K460

^a^5’NC: 5’ non coding region

^b^3’NC: 3’ non coding region

^c^not applicable
