# Supplementary material for: Distribution of Barley yellow dwarf virus-PAV in the Sub-Antarctic Kerguelen Islands and Characterization of Two New Luteovirus Species
Source: PLoS One. 2013 Jun 18;8(6):e67231. doi: 10.1371/journal.pone.0067231 (PMC3688969; doi:10.1371/journal.pone.0067231)
Supplement: Table S6 — Percentage of amino acid divergence observed between the various genomic regions of BYDV-Ker-II K465 and members of Luteoviridae family. (DOCX) [file pone.0067231.s006.docx]

**Table S6.** Percentage of amino acid divergence observed between the various genomic regions of BYDV-Ker-II K465 and members of *Luteoviridae* family

| **Virus name and accession number** | **5’NC^a^(154 nt)** | **P1 (339 aa)** | **P1-P2 (867 aa)** | **P3 (196 aa)** | **P3-P5 (672 aa)** | **P4 (152aa)** | **P6 (108 aa)** | **3’NC(3’ partial 607 nt)^b^** |
| --- | --- | --- | --- | --- | --- | --- | --- | --- |
| BYDV-PAV-I EF521849 | 37.7 | 50.3 | 33.9 | 34.2 | 39.5 | 35.5 | 69.2 | 47.3 |
| BYDV-PAV-II EU332309 | 38.0 | 50.9 | 34.1 | 33.7 | 39.8 | 34.9 | 66.7 | 47.1 |
| BYDV-PAV-III EU332318 | 39.7 | 49.7 | 33.7 | 36.4 | 40.9 | 32.9 | 69.4 | 48.4 |
| BYDV-MAV D11028 | 41.7 | 50.3 | 33.8 | 24.5 | 40.0 | 28.3 | 77.8 | 36.9 |
| BYDV-GAV EU402386 | 39.1 | 49.4 | 33.8 | 24.0 | 38.9 | 27.0 | 69.4 | 46.4 |
| BYDV-GPV L10356 | na ^c^ | na ^c^ | na ^c^ | 57.1 | na ^c^ | 76.8 | na ^c^ | na ^c^ |
| BYDV-SGV U06866 | na ^c^ | na ^c^ | na ^c^ | 40.3 | 53.4 | 39.0 | na ^c^ | na ^c^ |
| BYDV-RMV Z14123 | na ^c^ | na ^c^ | na ^c^ | 55.0 | na ^c^ | 71.4 | na ^c^ | na ^c^ |
| BYDV-Ker-III K460 KC559092 | na ^c^ | 42.8 | 28.4 | 34.7 | 40.1 | 32.2 | na ^c^ | na ^c^ |
| BYDV-Ker-II K439 KC571999 | 14.4 | 34.3 | 20.0 | 5.1 | 13.0 | 7.9 | 22.2 | 6.7 |
| BLRV NC003369 | 62.8 | 69.1 | 49.7 | 63.8 | 71.0 | 72.4 | na ^c^ | 66.0 |
| RSDaV EU024678 | 63.0 | 62.9 | 46.8 | 58.5 | 71.5 | 75.8 | 94.4 | 58.1 |
| SbDV JN674402 | 59.5 | 71.9 | 50.8 | 60.4 | 68.2 | 71.7 | na ^c^ | 64.9 |
| PEMV-1 NC003629 | 73.4 | 85.0 | 92.0 | 74.3 | 69.0 | na ^c^ | na ^c^ | 72.0 |
| BChV NC002766 | 73.9 | 88.8 | 89.3 | 54.9 | 69.2 | 75.2 | na ^c^ | 64.8 |
| PLRV NC001747 | 64.0 | 92.0 | 90.9 | 58.5 | 70.7 | 72.9 | na ^c^ | 68.8 |

^a^5’NC: 5’ non coding region. Percentage of nucleotide divergence

^b^3’NC: 3’ non coding region. Percentage of nucleotide divergence

^c^not applicable
