# Supplementary material for: Distribution of Barley yellow dwarf virus-PAV in the Sub-Antarctic Kerguelen Islands and Characterization of Two New Luteovirus Species
Source: PLoS One. 2013 Jun 18;8(6):e67231. doi: 10.1371/journal.pone.0067231 (PMC3688969; doi:10.1371/journal.pone.0067231)
Supplement: Table S7 — Percentage of amino acid divergence observed between the various genomic regions of BYDV-Ker-II K439 and members of Luteoviridae family. (DOCX) [file pone.0067231.s007.docx]

**Table S7.** Percentage of amino acid divergence observed between the various genomic regions of BYDV-Ker-II K439 and members of *Luteoviridae* family

| **Virus name and accession number** | **5’NC (partial 5’ 140 nt) ^a^** | **P1 (339aa)** | **P1-P2 (867aa)** | **P3 (196 aa)** | **P3-P5 (666 aa)** | **P4 (152aa)** | **P6 (108aa)** | **3’NC(3’ partial 605 nt)^b^** |
| --- | --- | --- | --- | --- | --- | --- | --- | --- |
| BYDV-PAV-I EF521849 | 37.1 | 48.1 | 34.2 | 33.2 | 39.3 | 34.9 | 61.5 | 48.8 |
| BYDV-PAV-II EU332309 | 39.5 | 48.4 | 34.3 | 32.7 | 39.6 | 34.2 | 61.1 | 48.3 |
| BYDV-PAV-III EU332318 | 38.7 | 47.5 | 33.3 | 36.9 | 40.8 | 30.3 | 66.7 | 48.5 |
| BYDV-MAV D11028 | 36.9 | 47.8 | 34.1 | 23.5 | 38.7 | 28.9 | 77.8 | 37.5 |
| BYDV-GAV EU402386 | 37.9 | 47.2 | 33.9 | 23.0 | 38.1 | 27.6 | 69.4 | 47.9 |
| BYDV-GPV L10356 | na ^c^ | na ^c^ | na ^c^ | 58.1 | na ^c^ | 75.4 | na ^c^ | na ^c^ |
| BYDV-SGV U06866 | na ^c^ | na ^c^ | na ^c^ | 38.8 | 53.0 | 39.0 | na ^c^ | na ^c^ |
| BYDV-RMV Z14123 | na ^c^ | na ^c^ | na ^c^ | 55.6 | na ^c^ | 72.1 | na ^c^ | na ^c^ |
| BYDV-Ker-III K460 KC559092 | na ^c^ | 28.3 | 22.0 | 34.7 | 39.2 | 32.9 | na ^c^ | na ^c^ |
| BYDV-Ker-II K465 KC572000 | 14.4 | 34.3 | 20.0 | 5.1 | 13.0 | 7.9 | 22.2 | 6.7 |
| BLRV NC003369 | 63.0 | 68.8 | 49.3 | 64.9 | 71.9 | 74.1 | na ^c^ | 65.9 |
| RSDaV EU024678 | 63.6 | 61.4 | 47.2 | 57.9 | 72.4 | 75.2 | 97.2 | 54.8 |
| SbDV JN674402 | 57.8 | 69.2 | 49.0 | 60.9 | 67.3 | 72.4 | na ^c^ | 63.9 |
| PEMV-1 NC003629 | 69.3 | 83.8 | 90.5 | 75.4 | 70.3 | na ^c^ | na ^c^ | 74.3 |
| BChV NC002766 | 72.7 | 87.9 | 89.4 | 56.5 | 69.1 | 77.2 | na ^c^ | 64.0 |
| PLRV NC001747 | 62.8 | 93.2 | 90.0 | 58.5 | 70.1 | 70.7 | na ^c^ | 66.7 |

^a^5’NC: 5’ non coding region

^b^3’NC: 3’ non coding region

^c^not applicable
